# A 2026 Outbreak of *Locusta migratoria* (Orthoptera: Acrididae) in Korea: Evidence of Partial Gregarization from Sex-Specific Morphometrics

**DOI:** 10.3390/insects17101041

**Published:** 2026-10-10

**Authors:** Sumin Oh, Woong Choi, Kwang-Ho Kim, Sung-Wook Jeon, Jongho Park, Boyoon Seo, Min Hyeuk Lee

**Affiliations:** 1 Pests and Weeds Control Division, National Institute of Agricultural Sciences, Wanju 55365, Republic of Korea; suminoh@korea.kr (S.O.);; 2 Graduate School of Plant Protection and Quarantine, Jeonbuk National University, Jeonju 54896, Republic of Korea

**Keywords:** *Locusta migratoria*, phase polyphenism, phenotypic plasticity, sexual dimorphism, reference calibration, population monitoring

## Abstract

The migratory locust, *Locusta migratoria*, exhibits density-dependent phase polyphenism. In June 2026, a large outbreak occurred on reclaimed agricultural land in Goheung, Republic of Korea. We quantified adult morphometric phase status using Korean general-reference and laboratory-crowded populations. Six body dimensions were measured in 173 adults comprising 47 general-reference, 46 laboratory-crowded, and 80 Goheung individuals. The ratios of hind femur length to head width (F/C) and forewing length to hind femur length (E/F) showed strong discrimination between the reference groups and were used to establish sex-specific classification boundaries. Of the 80 Goheung adults, 23 (28.8%) were classified as gregarious-like, 47 (58.8%) as transitional, and 10 (12.5%) as solitarious-like. In contrast, all 28 fifth-instar nymphs examined showed conspicuous coloration associated with gregarization. These findings indicate partial morphometric gregarization in the adult population and show that gregarization-associated nymphal coloration did not correspond to uniformly gregarious adult morphology in this outbreak. This study provides the first sex-specific morphometric assessment of a Korean outbreak population of *L. migratoria* and a preliminary framework for monitoring adult phase composition using locally calibrated criteria.

## Simple Summary

The migratory locust, *Locusta migratoria*, can change its coloration, behavior, and body proportions as population density increases. In June 2026, a large outbreak occurred on reclaimed agricultural land in Goheung, Republic of Korea. We compared adult body measurements from this outbreak with those of Korean reference specimens and locusts maintained under crowded conditions. Because males and females differed in their body proportions, we developed separate classification criteria for each sex. Of the 80 outbreak adults examined, 58.8% were classified as intermediate, 28.8% showed characteristics associated with crowding, and 12.5% showed characteristics associated with solitary living. Although all 28 immature locusts examined had conspicuous coloration associated with crowding, most adults had intermediate body proportions. This contrast suggests that immature coloration alone may not fully reflect the extent of crowding-related changes in adult body proportions. These findings provide a starting point for monitoring crowding-related changes in adult locusts using body measurements and separate criteria for males and females developed from Korean populations.

## 1. Introduction

The migratory locust, *Locusta migratoria* (Linnaeus, 1758), is widely distributed across Eurasia, Africa, and Oceania and is a classic example of density-dependent phase polyphenism. As population density changes, the behavior, coloration, physiology, reproduction, and morphology can shift reversibly between the solitarious and gregarious phase states [1,2,3,4]. At low density, individuals are generally dispersed and display solitarious characteristics, whereas increasing density promotes contact, aggregation, and the development of gregarious traits. Across its range in Africa, Asia, and parts of Europe, *L. migratoria* can form large swarms of gregarious adults that migrate over long distances [5,6,7,8] and cause extensive damage to cereal crops [9]. Phase change is not an instantaneous binary switch; different traits respond at different rates, and natural populations commonly contain transitional individuals between the two extremes [4]. Assessing phase-related traits can therefore complement population-density measurements when characterizing an outbreak [4].

Adult morphometric phase state has traditionally been assessed using the ratio of hind femur length to maximum head width (F/C) and the ratio of forewings (tegmina) length to hind femur length (E/F). Gregarization is generally associated with a lower F/C and a higher E/F. These directional changes have been reproduced experimentally under different rearing densities and after neuroendocrine manipulation, supporting the biological relevance of both ratios as phase-related traits [10,11]. Using geographically diverse material compiled by Farrow and Colless [12], Tanaka and Zhu [5] summarized population-level reference ranges of F/C 2.85–3.14 and E/F 1.95–2.19 for gregarious locusts and F/C 3.01–3.94 and E/F 1.68–1.91 for solitarious locusts. These values are useful external references but were not optimized as individual-level diagnostic thresholds, and the published F/C ranges overlap. In addition, sexual dimorphism in F/C and E/F has been reported in outbreak populations from China and Japan [5,13], while geographic and phylogenetic background can affect F/C ratios [14]. Consequently, a single sex-independent reference range may be insufficient for individual phase assessment in a geographically distinct population.

Large outbreaks of *L. migratoria* are rare in Korea. Historical records include an event in 1604 that has been interpreted as a probable migratory-locust outbreak [15], and a major outbreak was documented in the reclaimed lands of Haenam, Jeollanam-do, in 2014 [16]. During the 2014 Haenam outbreak, nymphs with conspicuous coloration occurred at high densities, but prompt control measures by the authorities prevented these densities from persisting into the adult stage, and no mass aggregations of adults were confirmed. Mitochondrial analyses showed that the sampled Haenam locusts belonged to the Northern lineage [16,17].

A recent outbreak of migratory locusts was observed in coastal reclaimed land of Goheung, Jeollanam-do in June 2026. The present study aimed to characterize the morphometric phase state of this outbreak population while avoiding direct transfer of geographically heterogeneous reference ranges. We therefore (i) screened 15 morphometric ratios in Korean general-reference and laboratory-crowded adults, (ii) selected phase indicators on the basis of statistical performance, biological interpretation, and comparability with previous locust studies, (iii) derived reproducible sex-specific operational boundaries using only the two reference groups, and (iv) applied those boundaries independently to the Goheung outbreak population. Mitochondrial cytochrome c oxidase subunit I (COI) sequences of the Goheung population were additionally used to confirm species identity.

## 2. Materials and Methods

### 2.1. Field Survey and Sampling

An initial field survey was conducted on 24 June 2026 in Goso-ri, Goheung-eup, Goheung-gun, Jeollanam-do, Republic of Korea (34.614170° N, 127.223918° E), followed by a second survey on 28 June 2026. The outbreak occurred in reclaimed agricultural land. During the surveys, land use, crop condition, developmental stages present, spatial distribution of locusts, and visible crop damage were recorded. During the two field surveys in Goheung, we collected 80 adults (51 females and 29 males) and 28 fifth-instar nymphs using a sweep net. Adults were used for morphometric analysis, and nymphs were examined for coloration associated with gregarization. Adult specimens were killed by freezing and subsequently pinned and dried for morphometric measurements. Voucher material was deposited in the Agricultural Insect Collection of the National Institute of Agricultural Sciences.

### 2.2. Reference Populations and Morphometric Measurements

The general-reference group comprised 47 adult specimens of *Locusta migratoria* from Korea (18 females and 29 males) preserved in the insect collection of the National Institute of Agricultural Sciences. Specimens were collected primarily between 1958 and 1998 from Suwon, Taean, Onyang, and Jeju, together with 10 adults from Muan, Jeollanam-do, on 19 September 2015. These specimens were selected to represent general Korean material unrelated to documented outbreak events.

The laboratory-crowded reference comprised 46 adults (26 females and 20 males) obtained from a commercial culture originating from Icheon, Gyeonggi-do. More than 5000 locusts were maintained in an approximately 15 m^2^ rearing space. Because rearing density and its history across generations were not experimentally controlled, this group was used as a crowded reference without assuming full gregarization.For each adult, maximum forewing length (E), maximum hind femur length (F), maximum head width (C), hind tibia length (T), pronotum length (P), and total body length (L) were measured to 0.01 mm using a Mitutoyo digital caliper (Mitutoyo Corp., Kawasaki, Japan). All measurements were taken by the first author (S. Oh) using the same anatomical landmarks, equipment, and measurement protocol (Supplementary Figure S2). If a body part was missing or damaged, only ratios requiring that measurement were treated as missing. The final morphometric dataset comprised 173 adults.

### 2.3. Morphometric Ratios and Statistical Screening

From the six linear measurements, 15 non-redundant ratios were calculated: E/F, E/C, E/T, E/P, E/L, F/C, F/T, F/P, F/L, T/C, T/P, T/L, P/C, P/L, and C/L. Indicator screening was performed separately by sex using only the general-reference and laboratory-crowded groups; the Goheung population was excluded from all indicator selection and threshold calibration. For each ratio, discrimination between the two reference groups was quantified using the area under the receiver operating characteristic curve (ROC-AUC). Because the direction of phase-related change differs among ratios, separation AUC was defined as max (AUC, 1 − AUC). A 95% confidence interval was estimated by 1000 stratified bootstrap resamples of the two reference groups (seed = 20260821). All 105 pairwise combinations of the 15 ratios were additionally evaluated by linear discriminant analysis (LDA) with leave-one-out cross-validation (LOOCV). Performance was summarized by accuracy, balanced accuracy, cross-validated AUC (CV-AUC), sensitivity, and specificity. Because many ratios share component measurements and are not mathematically independent, the two-ratio LDA results were used only for exploratory screening.

Sex differences in F/C and E/F were evaluated within each group using two-sided Mann–Whitney U tests. Group differences among the general reference, Goheung outbreak, and laboratory-crowded populations were tested separately by sex using Kruskal–Wallis tests followed, where appropriate, by three pairwise Mann–Whitney U tests with Bonferroni adjustment. Epsilon-squared (ε^2^) and rank-biserial correlation were reported as effect-size measures. Full statistical outputs are provided in the Supplementary Material.

### 2.4. Derivation of Sex-Specific Operational Phase Criteria

The two morphometric ratios, F/C and E/F, were retained as the final phase indicators because both showed high discrimination in each sex, their phase-related directional responses are experimentally established, and they permit direct comparison with the classical locust literature. Sex-specific operational boundaries were derived using the general-reference and laboratory-crowded groups only.

For each sex and ratio, candidate cutoffs were defined as midpoints between consecutive distinct observations, and the cutoff maximizing Youden’s J statistic (sensitivity + specificity − 1) was identified. The two reference groups were then resampled separately with replacement to their original sample sizes, and the optimal cutoff was recalculated for 1,000,000 stratified bootstrap replicates (seed = 20260821). The 2.5th and 97.5th percentiles of the bootstrap optimal-cutoff distribution were used to define a conservative grey zone.

Because lower F/C and higher E/F indicate the gregarious direction, males were classified as gregarious-like when F/C ≤ 3.367 and E/F ≥ 1.948, and as solitarious-like when F/C ≥ 3.481 and E/F ≤ 1.933. For females, the corresponding criteria were F/C ≤ 3.196 and E/F ≥ 1.985 for gregarious-like individuals and F/C ≥ 3.292 and E/F ≤ 1.958 for solitarious-like individuals. An individual was assigned to an extreme morphometric class only when both ratios supported the same direction; all other individuals were classified as transitional. Monte Carlo sensitivity to the random seed was evaluated using five seeds, each with 100,000 bootstrap replicates. These criteria are Korean reference-calibrated operational boundaries rather than universal diagnostic cutoffs; gregarious-like and solitarious-like denote morphometric resemblance to the laboratory-crowded and general-reference groups, respectively, rather than independently verified phase states.

For external comparison only, specimens were also evaluated against the population-level ranges summarized by Tanaka and Zhu [5] from the geographically diverse material of Farrow and Colless [12]: F/C 2.85–3.14 and E/F 1.95–2.19 for gregarious populations, and F/C 3.01–3.94 and E/F 1.68–1.91 for solitarious populations. These values were not used to derive or modify the Korean operational criteria. Membership in the two-dimensional published ranges is reported as a supplementary exploratory comparison.

### 2.5. COI Sequencing and Identification

DNA was extracted from five Goheung adults using the DNeasy Blood & Tissue Kit (QIAGEN, Hilden, Germany) following the manufacturer’s protocol. A COI fragment was amplified using LCO1490 and HCO2198 [18]. PCR conditions were 94 °C for 5 min; 37 cycles of 94 °C for 1 min, 45.2 °C for 30 s, and 72 °C for 1 min; and a final extension at 72 °C for 5 min. PCR products were sequenced by Macrogen Inc. (Seoul, Republic of Korea). Forward and reverse chromatograms were assembled into consensus sequences and manually inspected. Consensus sequences were compared with GenBank records using BLASTn. Newly generated sequences were deposited in GenBank under accession numbers QB017441-QB017445.

### 2.6. Software and Reproducibility

Morphometric analyses were performed in Python 3.13.5 using pandas 2.2.3, NumPy 2.3.5, SciPy 1.17, and scikit-learn 1.8.0. ABI chromatograms were inspected and processed using Biopython 1.86 for sequence-quality assessment and bidirectional alignment. The final raw morphometric data, analysis results, and code are supplied as Supplementary Materials. Generative AI tools were used to assist with statistical-code drafting and figure visualization.

## 3. Results

### 3.1. Outbreak Observations and Identification

**Figure 1:**
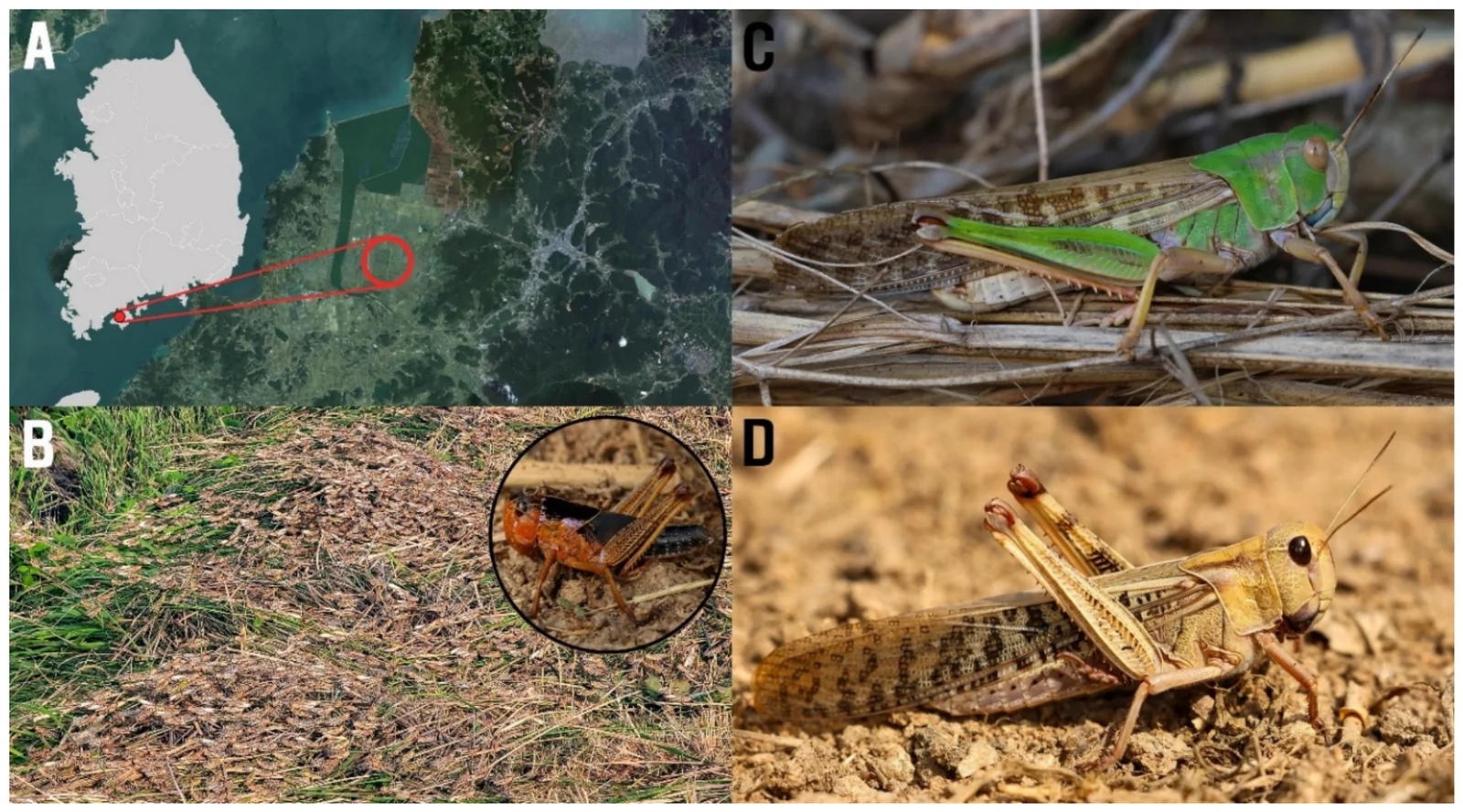
Study area and representative *Locusta migratoria* observed during the 2026 Goheung outbreak. (**A**) Location of the outbreak area in Goheung, southern Republic of Korea. (**B**) Dense field occurrence, with a fifth-instar nymph showing conspicuous dark and orange coloration in the inset. (**C**,**D**) Representative adult phenotypes observed during the survey. Map data were provided by the National Geographic Information Institute, Republic of Korea.

In June 2026, a high-density occurrence of adult *Locusta migratoria* was confirmed in reclaimed agricultural fields in Goso-ri, Goheung-gun, Jeollanam-do, Republic of Korea (Figure 1A). According to a government survey report, the outbreak affected approximately 100 ha of reclaimed agricultural fields and adjacent farm roads. Adults and late-instar nymphs co-occurred within the outbreak area, and aggregations of adults were observed on host plants at some sites (Figure 1B). Adult coloration ranged from green (Figure 1C) to yellowish brown (Figure 1D), with brown forms predominating, whereas all 28 fifth-instar nymphs examined showed conspicuous black markings and orange-to-yellow coloration associated with gregarization (Figure 1B, inset). The affected fields had been cultivated with Italian ryegrass (*Lolium multiflorum*) and were largely in a post-harvest stage before the sowing of forage barnyard millet.

COI analysis of five Goheung adults identified three haplotypes. In BLASTn comparisons with *L. migratoria* records, H1 and H3 matched KY199448.1 and AB497225.1, respectively, with 100% identity, whereas H2 showed 99.85% identity to AB497225.1. Across the shared 563-bp region, H1 and H3 showed 100% identity to sequences from the Haenam outbreak population (KY174968.1-KY174979.1), whereas H2 differed by a single nucleotide from its closest Haenam matches (99.82% identity; Supplementary Table S10).

### 3.2. Discriminatory Performance of Morphometric Ratios

Several ratios strongly separated the general-reference and laboratory-crowded groups, but their rankings differed between sexes (Supplementary Table S1). In females, E/F and F/L each achieved a separation AUC of 1.000, followed by F/C (0.970), E/T (0.946), and T/L (0.939). In males, F/C and F/L each achieved an AUC of 0.981, followed by T/C (0.938), E/F (0.929), and T/L (0.843).

The best two-ratio LOOCV-LDA models also differed by sex (Supplementary Table S2). Several E/F-containing combinations achieved a balanced accuracy of 1.000 in females. In males, E/T + F/L achieved a balanced accuracy of 0.982 (CV-AUC = 0.991), while E/C + F/L achieved 0.975 (CV-AUC = 0.997). The classical E/F + F/C combination yielded an accuracy of 0.977, balanced accuracy of 0.972, and CV-AUC of 0.996 in females, and 0.939, 0.933, and 0.978, respectively, in males. Thus, F/C and E/F were not statistically unique optimum variables, but they retained strong discriminatory performance in both sexes.

### 3.3. Morphometric Phase Variation in the Goheung Outbreak Population

F/C and E/F showed clear sexual differences. In the general-reference group, male F/C was significantly higher than female F/C (3.679 ± 0.148 vs. 3.425 ± 0.139; Mann–Whitney U = 473, *p* < 0.001), and the same direction was observed in the laboratory-crowded group (3.197 ± 0.155 vs. 3.084 ± 0.141; U = 361, *p* = 0.026). Female E/F exceeded male E/F in the laboratory-crowded group (2.128 ± 0.122 vs. 2.026 ± 0.075; U = 103, *p* < 0.001), whereas the sex difference in E/F was not significant in the general-reference group (*p* = 0.123). Both F/C and E/F differed significantly between sexes in the Goheung population (Supplementary Table S4b).

**Figure 2:**
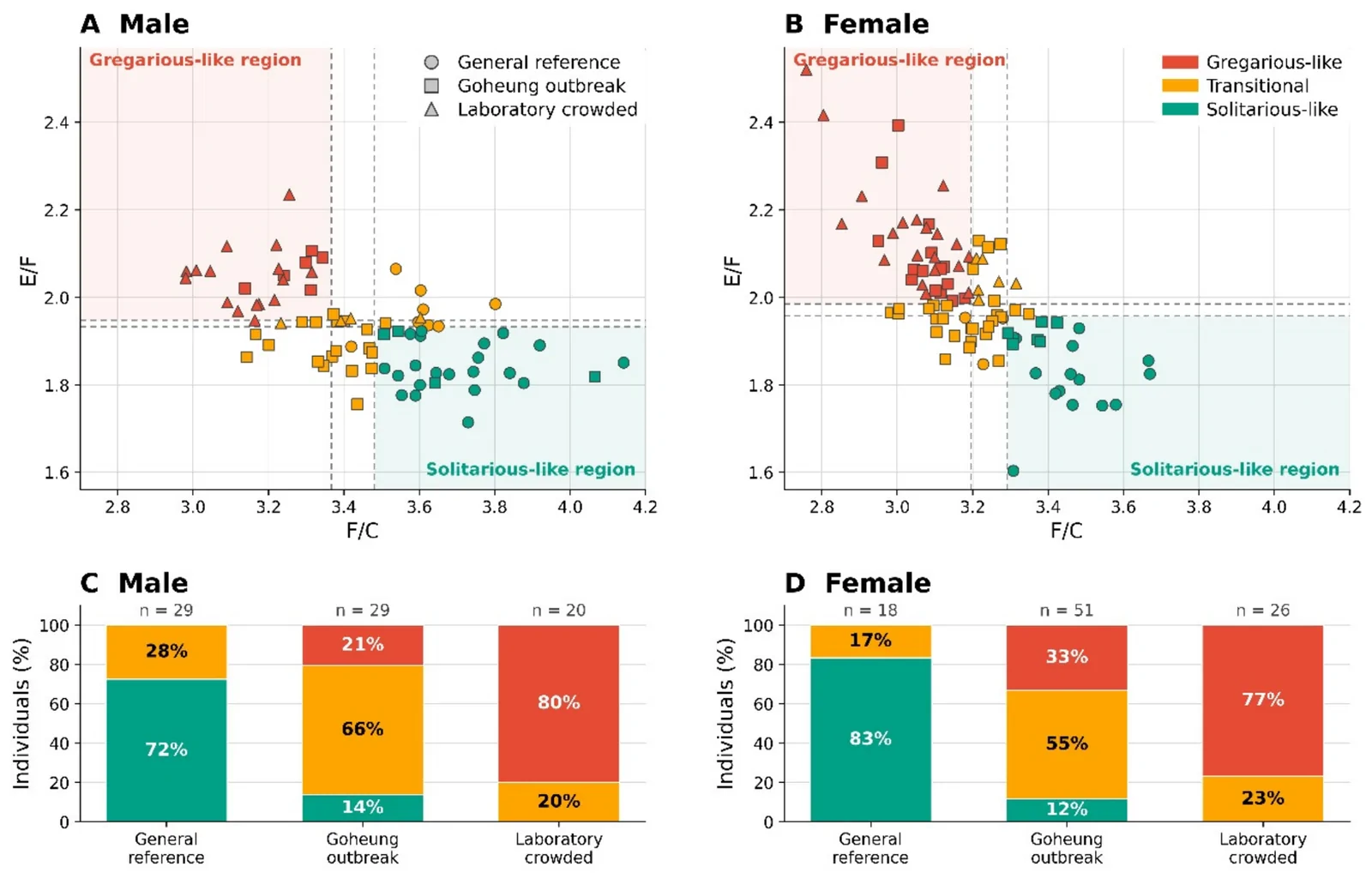
Sex-specific morphometric phase assessment of adult *Locusta migratoria*. (**A**,**B**) F/C–E/F distributions for males and females. Colors indicate operational phase class and symbols indicate sample group; dashed boundaries and shaded regions show the sex-specific gregarious-like and solitarious-like criteria. (**C**,**D**) Phase-class composition of the general-reference, Goheung outbreak, and laboratory-crowded groups. Values above bars indicate sample sizes. Percentages were rounded to the nearest whole number.

Goheung adults were generally intermediate between the two reference groups, although the magnitude of the shift depended on sex and ratio (Supplementary Tables S3 and S4; Figure 2A,B). Male F/C declined stepwise from the general reference (3.679 ± 0.148) to Goheung (3.392 ± 0.177) and the laboratory-crowded group (3.197 ± 0.155), with significant differences among all three groups (Kruskal–Wallis H = 51.07, *p* < 0.001, ε^2^ = 0.654). Female E/F similarly increased from 1.832 ± 0.087 to 2.001 ± 0.104 and 2.128 ± 0.122, respectively (H = 52.38, *p* < 0.001, ε^2^ = 0.548). By contrast, male E/F did not differ significantly between Goheung and the general reference after Bonferroni adjustment (adjusted *p* = 0.125), and female F/C did not differ significantly between Goheung and the laboratory-crowded group (adjusted *p* = 0.070).

The reference-derived Youden cutoffs were 3.419 for male F/C (J = 0.950), 1.939 for male E/F (J = 0.828), 3.227 for female F/C (J = 0.868), and 1.975 for female E/F (J = 1.000). Their 1,000,000-replicate bootstrap 95% percentile intervals were 3.367–3.481, 1.933–1.948, 3.196–3.292, and 1.958–1.985, respectively (Supplementary Table S5). When the resulting grey-zone criteria were reapplied to the reference populations, no general-reference individual was classified as gregarious-like and no laboratory-crowded individual was classified as solitarious-like. Overall, 36 of 47 general-reference adults (76.6%) were solitarious-like, and 36 of 46 laboratory-crowded adults (78.3%) were gregarious-like; the remaining reference adults were transitional.

Among the 29 Goheung males, 6 (20.7%) were gregarious-like, 19 (65.5%) transitional, and 4 (13.8%) solitarious-like. Among 51 females, 17 (33.3%) were gregarious-like, 28 (54.9%) transitional, and 6 (11.8%) solitarious-like. Combined, 23 of 80 adults (28.8%) were gregarious-like, 47 (58.8%) transitional, and 10 (12.5%) solitarious-like (Figure 2C,D; Supplementary Tables S7 and S8). Across five random-seed sensitivity analyses, reference cross-extreme errors remained zero and the Goheung transitional proportion remained between 57.5% and 60.0% (Supplementary Table S6).

Application of the previously published two-dimensional reference ranges produced substantially lower range membership in the Korean reference groups than did the locally calibrated operational classification, particularly in the laboratory-crowded group (Supplementary Figure S1 and Supplementary Table S9). Only 35.0% of laboratory-crowded males and 46.2% of laboratory-crowded females fell within the published gregarious range, and 58.6% of general-reference males and 77.8% of general-reference females fell within the published solitarious range. In Goheung, published-range membership differed markedly between sexes.

## 4. Discussion

In this study, we report an outbreak of *Locusta migratoria* in Goheung, Korea, and quantify phase-related morphometric characteristics of Korean adults. During the previous outbreak in Haenam in 2014, high densities of nymphs with gregarious-associated coloration were recorded, but adult phase status was not quantitatively assessed [16]. The COI haplotypes identified in Goheung adults were identical or closely similar to those reported from Haenam, although the short COI fragment analysed here does not establish direct population continuity or a common outbreak origin. The occurrence of both outbreaks on reclaimed agricultural land raises the possibility that cultivation conditions and large-scale cultivation of monocotyledonous crops in these habitats may be associated with locust outbreaks and gregarization in Korea [19].

Morphometric phase assessment indicated an intermediate phase composition in the Goheung adult population sampled in June 2026, with 58.8% of adults classified as transitional, 28.8% as gregarious-like, and 12.5% as solitarious-like. We, therefore, interpret the sampled population as showing partial morphometric gregarization rather than a uniformly gregarious adult morphology. Intermediate or transient forms between the solitarious and gregarious extremes are a recognized component of locust phase polyphenism [4,20]. The predominance of the transitional morphology among adults contrasted with the conspicuous gregarious-associated coloration observed in all 28 fifth-instar nymphs examined during the same survey period. Phase-related coloration and morphometric characteristics do not necessarily respond at the same rate or during the same developmental period [4,20]. Thus, nymphal coloration alone was insufficient to assess the extent of adult morphometric gregarization in this outbreak. Similarly, field populations in Japan have included predominantly solitarious-like adults alongside individuals with lower F/C and higher E/F values associated with gregarization [13].

Sex should also be considered when assessing adult morphometry. Consistent with previous observations [5,13], significant sex differences in both F/C and E/F were detected in the Goheung population. Screening all 15 pairwise ratios showed that F/C and E/F were not uniquely optimal, although both maintained strong discriminatory performance in males and females. Their established use as classical indices of locust phase morphology, experimentally demonstrated responses to density-related phase change, and comparability with previous studies make them useful indicators for morphometric phase assessment [5,11,13,21].

The limited correspondence between published F/C–E/F ranges and the Korean reference populations highlights the need for caution when applying these ranges directly to individual locusts. Only 35.0% of laboratory-crowded males and 46.2% of laboratory-crowded females fell within the previously published gregarious range. Earlier studies have demonstrated geographical or strain-related variation in the morphometric traits of *L. migratoria* [12,14,22], further supporting the consideration of local reference populations when establishing classification criteria. 

A recent study highlighted the predominance of laboratory studies and the limited integration of field evidence in locust phase research [21]. Our results extend the classical F/C–E/F framework by deriving sex-specific thresholds from Korean general-reference and laboratory-crowded populations and incorporating bootstrap-estimated uncertainty as a grey zone. We then applied these criteria to the Goheung outbreak population, which was not used in threshold calibration, to quantify morphometric phase composition in a field population. Individuals with ambiguous or discordant values were classified as transitional rather than forced into either phase extreme; this category does not itself indicate an ongoing phase transition. The unstandardized density history of the laboratory-crowded reference may affect the position of the estimated cutoffs and the resulting class proportions. The correspondence of these morphometric categories to behavioral and physiological phase traits remains to be validated in independent populations. The supplied code can be applied to other *L. migratoria* populations with comparable measurements and suitable local reference groups, but sex-specific cutoffs and grey zones require local recalibration.

## 5. Conclusions

We report the first documented mass occurrence of adult *Locusta migratoria* in Korea and identify partial morphometric gregarization in the Goheung outbreak using sex-specific criteria. Nymphal coloration alone was insufficient to assess the extent of adult morphometric gregarization in this outbreak. Applying these criteria to other populations requires recalibration using suitable local reference groups.

## Data Availability

The raw morphometric data, analysis results, and Python code are provided in the Supplementary Materials. The COI sequences generated in this study have been deposited in GenBank under accession numbers QB017441-QB017445.

## Supplementary Materials

The following supporting information can be downloaded at: https://www.mdpi.com/article/10.3390/insects17101041/s1, Supplementary_Data.xlsx, containing the raw morphometric data and Supplementary Tables S1–S10; Supplementary_Figures.pdf, containing Figures S1 and S2; and Locusta_analysis_code.py, containing the Python code for the morphometric statistical analyses.
