# Supplementary material for: A 2026 Outbreak of Locusta migratoria (Orthoptera: Acrididae) in Korea: Evidence of Partial Gregarization from Sex-Specific Morphometrics
Source: Insects. 2026 Oct 10;17(10):1041. doi: 10.3390/insects17101041 (PMC13705141; doi:10.3390/insects17101041)
Supplement: Supplementary file 1 [file insects-17-01041-s001.zip › Supplementary_Figures.pdf]

Supplementary_Figures

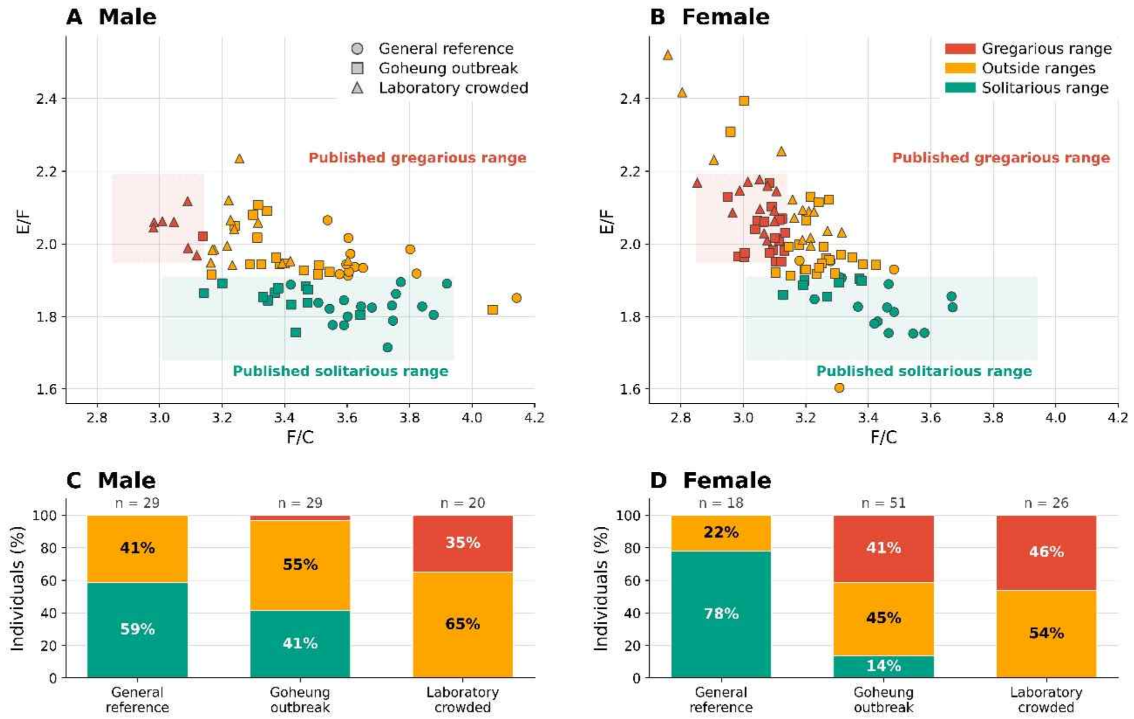

Figure S1. Comparison of F/C–E/F distributions with published international reference ranges. (A,B) Distributions of males and females; symbols indicate sample groups, and colors indicate membership in the published gregarious range, solitarious range, or neither. (C,D) Corresponding proportions by sex and group. Shaded areas show the ranges reported by Tanaka and Zhu (2005).

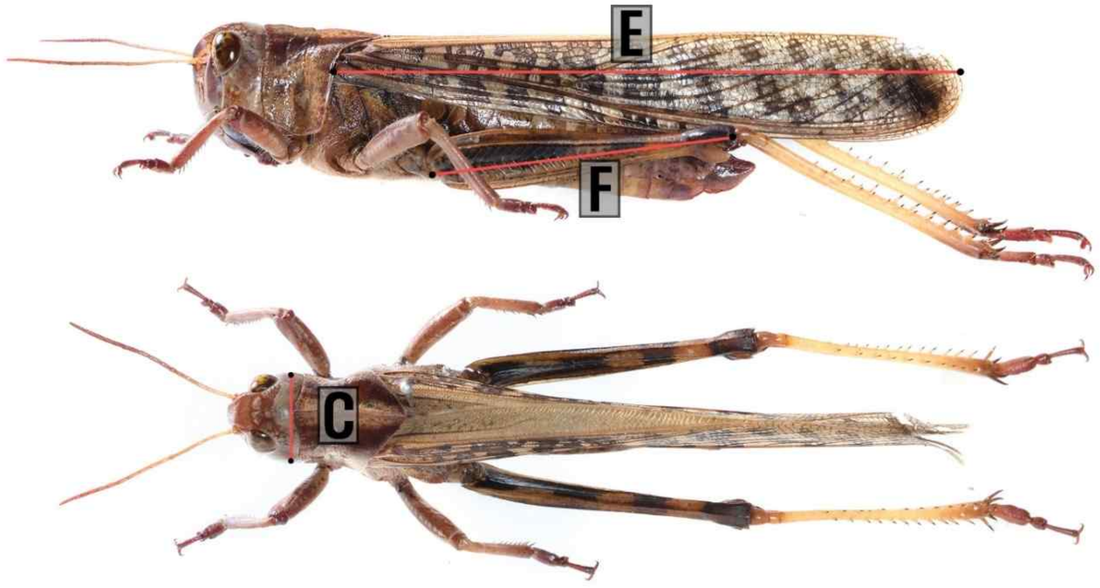

Figure S2. Morphometric measurements used for adult phase assessment.

E: maximum forewing length; F: maximum hind femur length; C: maximum head width. Red lines and black dots indicate the measurement axes and endpoints, respectively.
